# Prevalence of pain, analgesic self-medication and mental health in German pre-hospital emergency medical service personnel: a nationwide survey pilot-study

**DOI:** 10.1007/s00420-021-01730-x

**Published:** 2021-06-07

**Authors:** Luis Möckel, Angela Gerhard, Mara Mohr, Christoph Immanuel Armbrust, Christina Möckel

**Affiliations:** 1 HSD Hochschule Döpfer GmbH, University of Applied Sciences, Waidmarkt 3 & 9, 50676 Cologne, Germany; 2 HSD Hochschule Döpfer GmbH, University of Applied Sciences, Prüfeninger Straße 20, 93049 Regensburg, Germany; 3 Forschungszentrum Jülich, Wilhelm-Johnen-Straße, 52428 Jülich, Germany

**Keywords:** Musculoskeletal disease, Analgesics intake, Healthcare worker, Lower back issues

## Abstract

**Purpose:**

The aim of this study was to estimate the prevalence of pain, the extent of analgesics intake and the mental health status of German pre-hospital emergency medical service (EMS) personnel.

**Methods:**

We conducted a nationwide online survey, which consisted of sociodemographic and job-related items, questions on pain and analgesics intake and the short-version of the validated Depression–Anxiety–Stress Scale (DASS-21).

**Results:**

A total of 774 EMS personnel with a mean age of 33.03 (standard error [SE] 0.37) years were included into the final analysis of which 23.77% were female. Pain was reported by 58.64% (454 of 774) of the study participants with 10.72% (95% confidence interval [CI] 8.54%; 13.29%) suffering from chronic, 1.68% (95% CI 0.89%; 2.87%) from acute and 46.25% (95% CI 41.49%; 51.30%) from recurrent pain, respectively. Most frequent location of pain was lumbar spine. Analgesics were used by 52.76% (239 of 454) of pre-hospital EMS personnel with pain (acute 76.92% / chronic 69.88% / recurrent 47.90%). Moreover, participants with chronic and recurrent pain indicated significantly higher depression (*p* ≤ 0.001), anxiety (*p* ≤ 0.001), and stress (*p* ≤ 0.001) levels compared to those without pain, respectively.

**Conclusion:**

This study indicates a high prevalence of pain and analgesics usage in participating German pre-hospital EMS personnel and a poorer mental health in those with chronic and recurrent pain. Therefore, disease prevention and health promotion measures are needed to preserve health of pre-hospital EMS personnel.

## Introduction

People working in pre-hospital emergency medical service (EMS) are an essential group of skilled healthcare professionals, evaluating acute patients and providing first care (Ilper et al. 2014; Klepacka et al. 2018). It is also known that people working in pre-hospital EMS are often suffering from back complaints (Imani et al. 2018; Maguire et al. 2005; Okada et al. 2005; Zhang et al. 2019). A study with Chinese ambulance drivers indicated a 1-year prevalence of lower back pain lasting for at least 3 months of 12.3% (Zhang et al. 2019). Data from Imani et al. (2018) with 298 pre-hospital emergency technicians revealed that 46.3% experienced lower back pain of different intensities (Imani et al. 2018). In addition to lower back pain, Okada et al. (2005) reported feet, knee, shoulder and neck issues in 15.5–36.7% of EMS personnel. In contrast, less is known on the prevalence of gastrointestinal and head pain in EMS personnel, respectively. But in the general German population the prevalence of migraine is high with 14.8% in women and 6.0% in men (Porst et al. 2020). Still unknown is the extent of analgesic self-medication by German pre-hospital EMS personnel. For German nurses, a population likewise often affected by back pain, it was reported that they are more frequently using analgesics compared to other professionals (Techniker Krankenkasse 2019).

In addition to musculoskeletal diseases, paramedics or ambulance workers are more frequently diagnosed with mental disorders (Bennett et al. 2004; Berger et al. 2012; Sterud et al. 2006; Van Der Ploeg and Kleber 2003). Data from the Netherlands indicated that 8.6% of emergency personnel were at risk for burnout, whereas the general working population indicated a risk of 5.3% (Sterud et al. 2006; Van Der Ploeg and Kleber 2003). A large meta-regression analysis, including 28 studies with 20,424 rescuers calculated a worldwide prevalence of post-traumatic stress disorder (PTSD) of 10% and a European prevalence of 7.4% (Berger et al. 2012), respectively. When stratified by occupational group, ambulance workers indicated a higher PTSD prevalence (14.6%) compared to firefighters and police officers exposed to major disasters (Berger et al. 2012). In addition, a more recent study from New York revealed that daily occupational stressors and social conflicts were significantly associated with PTSD and depression symptom severity in EMS workers (Hruska and Barduhn 2021).

Latest numbers from 2018 reveal, that approximately 71,000 persons (31% female) are working in the pre-hospital emergency service in Germany. The majority of these persons were below 30 years of age (32%), followed by the age groups 30–39 years (28%), and 40–49 years (20%) (HPA 2021).

Pre-hospital EMS personnel represent a large group of persons working in the healthcare sector, which are vulnerable to pain and stress. Nevertheless, little is known about pain, mental health issues and in particular analgesic intake in this group in Germany. Therefore, the aim of this study was to estimate the prevalence of pain and the usage of analgesics in pre-hospital EMS personnel. Furthermore, we evaluated the type of pain (acute, chronic, recurrent) and the mental health status of the study participants.

## Methods

### Study design and participants

This study was a nationwide survey pilot-study with pre-hospital EMS personnel in Germany. The survey period lasted for 4 weeks in October and November 2020. Inclusion criteria were working in any position in the EMS and an age of 18 years or older. To approach EMS personnel, randomly selected emergency service stations in Germany were contacted. These stations were asked to distribute the link to the online survey to their EMS personnel. In addition, the Society for Promotion of Science in Emergency Services (*Gesellschaft zur Förderung der Wissenschaft im Rettungsdienst*) in Germany distributed the link to the survey.

### Questionnaire

The online survey consisted of sociodemographic as well as job-related questions on age, gender, trained EMS position and time working in EMS. Regarding pain, all study participants were asked to answer the following question, “Do you have any pain?” with the options to answer “No”, “Yes, acute pain", “Yes, chronic pain (defined as pain for 3 months or longer (Becker et al. 2013; Hensler et al. 2009))" or “Yes, recurrent pain". Recurrent pain was defined as repeating occurrence of pain, lasting for more than 24 h and pain-free episode(s) of at least one month (de Vet et al. 2002; Stanton et al. 2010). Only participants who claimed having pain were then asked to rate the severity of their pain, using a numeric rating scale with 0, equals to no pain, and 10, equals to strongest imaginable pain. In addition, participants with pain were also asked to define the location of pain and if they take any analgesics. Only participants who disclosed to use analgesics were asked to indicate the dosing frequency and the specific substance.

Finally, all study participants were asked to answer the short version of the Depression–Anxiety–Stress–Survey (DASS-21), which consists of 21 items (Henry and Crawford 2005; Lovibond and Lovibond 1995a, b; Lovibond and Lovibond 1995a, b; Nilges and Essau 2015). For this study, we used the validated German version of the DASS-21. The sub scales depression, anxiety and stress consist of seven items, respectively. To answer the questionnaire the study participants had to rate each item with 0, did not apply to me at all, 1, applied to me to some degree, or some of the time, 2, applied to me to a considerable degree, or a good part of time, 3, applied to me very much, or most of the time (Nilges and Essau 2015).

The survey was conducted in German language by using the online tool SoSci Survey (*SoSci *Survey 2020).

### Statistical analysis

Participants were included into the analysis if they answered the question on gender, age, EMS position, time working in emergency service and if they have pain. For presentation of sociodemographic and job-related characteristics proportion or means and standard error of the means (SE) were calculated. Prevalence of pain by type (acute, chronic and recurrent) was calculated for the overall study sample as well as by gender, and the corresponding 95% confidence intervals (CI) were computed.

Differences in frequency of pain between gender, by location of pain, and the analgesics intake by type of pain were analyzed using Pearson’s chi-squared test, respectively. To compare the severity of pain stratified by type of pain, ANOVA including post-hoc analysis with Tukey correction was used. For analysis of DASS-21 sub scales the respective items were summed up. Afterwards, sum of each score was multiplied by 2, to obtain doubled DASS-21 sub scale scores, which are equivalent to the DASS scores (Henry and Crawford 2005; Lovibond and Lovibond 1995a, b; Lovibond and Lovibond 1995a, b; Nilges and Essau 2015). ANOVAs as well as post-hoc analyses with Tukey correction were applied to compare doubled DASS-21 sub scale scores of pre-hospital EMS personnel by type of pain.

A *p* value of 0.05 was considered statistically significant and analysis was performed using JASP software package (JASP Team 2020).

## Results

### Characteristics of study participants

A total of 774 study participants with a mean age of 33.03 (SE 0.37) years and 23.77% females, were included into the final analysis (Table 1). The majority of study participants was working in the federal states Bavaria (19.51%), Rhineland-Palatinate (18.86%), and North Rhine-Westphalia (17.44%). A total of 89.14% of study participants were paramedics, 10.08% were EMS trainees and 0.78% were emergency physicians. Mean duration of working in EMS was 11.75 (SE 0.35) years and 30.10% stated to be smokers.

**Table 1:** Sociodemographic and job-related characteristics of study participants

Characteristics	*N* = 774
Gender	
% Female	23.77%
% Non-binary	0.52%
Age—mean (SE)	33.03 (0.37) years
Smokers—%	30.10%
Years working in EMS—mean (SE)	11.75 (0.35) years
Federal state—%	• Baden-Württemberg: 12.92%
	• Bavaria: 19.51%
	• Brandenburg: 0.90%
	• Berlin: 0.39%
	• Bremen: 0.26%
	• Hamburg: 1.03%
	• Hesse: 15.89%
	• Lower Saxony: 6.46%
	• Mecklenburg-Western Pomerania: 0.39%
	• Northrhine-Westphalia: 17.44%
	• Rhineland-Palatinate: 18.86%
	• Saarland: 0.90%
	• Saxony: 1.03%
	• Saxony-Anhalt: 1.16%
	• Schleswig–Holstein: 2.33%
	• Thuringia: 0.39%
EMS occupation	• Paramedic*: 89.14%
	• Emergency physician: 0.78%
	• Trainee: 10.08%

*Paramedic: includes all non-physician pre-hospital EMS occupations in Germany

### Prevalence and severity of pain

**Fig. 1:**
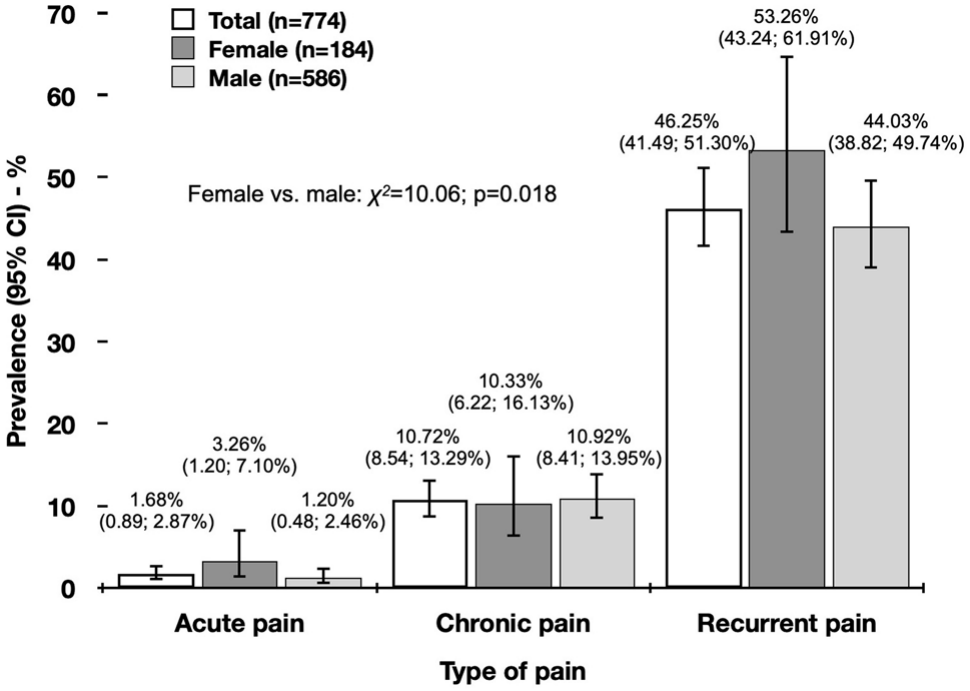
Prevalence of pain shown for full study sample and by gender

A total of 58.64% (454 of 774) pre-hospital EMS personnel indicated to have pain. Of these 10.72% (95% CI 8.54%; 13.29%) reported chronic pain, 1.68% (95% CI 0.89%; 2.87%) acute pain and 46.25% (95% CI 41.49%; 51.30%) recurrent pain, with significant differences between female and male (*χ*^*2*^ = 10.06; *p* = 0.018) (Fig. 1). Most frequent locations of pain (Table 2) were lumbar spine (66.67%), head (41.50%) and cervical spine (30.02%), with non-significant differences in frequency when stratified by type of pain (acute, chronic and recurrent). Nevertheless, frequency of pain at upper (*χ*^*2*^ = 8.71; *p* = 0.013) and lower (*χ*^*2*^ = 8.78; *p* = 0.012) limbs indicated significant differences between pain groups with highest number among EMS personnel with chronic pain (upper 27.71% / lower 32.53%) and lowest among those with recurrent pain (upper 14.29% / lower 17.93%), respectively.

**Table 2:** Location of pain in pre-hospital EMS personnel by type of pain; *P* values and χ^2^ for comparison between acute, chronic and recurrent pain

Location of pain	Total with pain (*n* = 453)	Acute pain (*n* = 13)	Chronic pain (*n* = 83)	Recurrent pain (*n* = 358)	*χ*^*2*^; *p* value
Lumbar spine	66.67%	61.54%	66.27%	66.95%	0.17; *p* = 0.917
Thoracic spine	26.93%	15.39%	25.30%	27.73%	1.11; *p* = 0.574
Cervical spine	30.02%	30.77%	37.35%	28.29%	2.63; *p* = 0.268
Lower limbs	20.75%	23.08%	32.53%	17.93%	8.78; *p* = 0.012
Upper limbs	16.78%	15.39%	27.71%	14.29%	8.71; *p* = 0.013
Head*	41.50%	30.77%	34.94%	43.42%	2.63; *p* = 0.269
Gastrointestinal	13.25%	15.59%	16.87%	12.33%	1.26; *p* = 0.532
Other location	4.64%	7.69%	7.23%	3.92%	1.95; *p* = 0.377

*Head pain combines headache, migraine and tension headache

Multiple answers were possible

Intensity of pain (*n* = 453) on the numerical pain scale indicated a severity of 4.00 (SE 0.07). The mean intensity of pain in EMS personnel showed significant differences between pain groups (*p* = 0.013). Post-hoc analysis revealed that study participants with chronic pain (*n* = 83; 4.41 [SE 0.17]) showed significantly (*p*_Tukey_ = 0.011) higher pain severities compared to those with recurrent pain (*n* = 357; 3.89 [SE 0.08]). Whereas severity of acute pain (*n* = 13; 4.23 [SE 0.55]) was comparable to severity of chronic (*p*_Tukey_ = 0.913) and recurrent pain (*p*_Tukey_ = 0.692), respectively.

### Analgesics intake

A total of 239 out of 454 (52.76%) pre-hospital EMS personnel with pain used analgesics and analgesics intake was significantly associated with type of pain (acute [76.92%]; chronic [69.88%]; recurrent [47.90%]; *χ*^*2*^ = 16.19; *p* ≤ 0.001). The mean frequency of analgesic intake was 2.32 (SE 0.16) times per week.

**Fig. 2:**
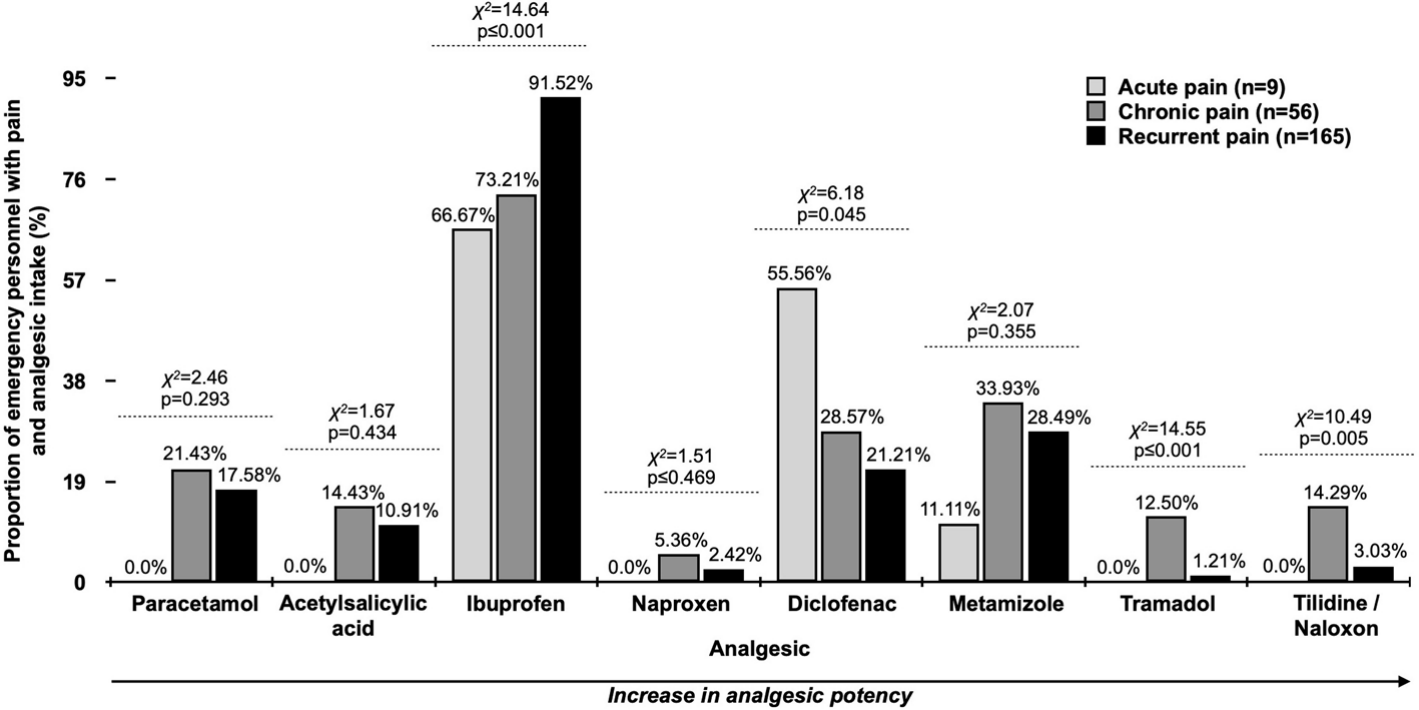
Most frequently used analgesics by pre-hospital EMS personnel who stated to have pain and to use analgesics. Multiple answers were possible

Most commonly mentioned analgesics (≥ 3.0%) by study participants (*n* = 239) were Ibuprofen (86.09%), Metamizole (29.13%), Diclofenac (24.35%), Paracetamol (17.83%), Acetylsalicylic acid (11.30%), Tilidine/Naloxone (5.65%), Tramadol (3.91%) and Naproxen (3.04%). When displayed by type of pain (Fig. 2), significant differences between pain groups were seen for intake of Ibuprofen (recurrent [91.52%]; chronic [73.21%]; acute [66.67%]; *χ*^*2*^ = 14.64; *p* ≤ 0.001). Diclofenac was more frequently used in participants with acute pain (55.56% vs. 28.57% chronic pain vs. 21.21% recurrent pain; *χ*^*2*^ = 6.18; *p* = 0.045). In addition, significant differences were also seen for intake of Tramadol (*χ*^*2*^ = 14.55; *p* ≤ 0.001) and Tilidine/Naloxone (*χ*^*2*^ = 10.49; *p* = 0.005) with highest proportion in EMS personnel with chronic pain, respectively.

### Depression, anxiety and stress level in pre-hospital EMS workers

**Fig. 3:**
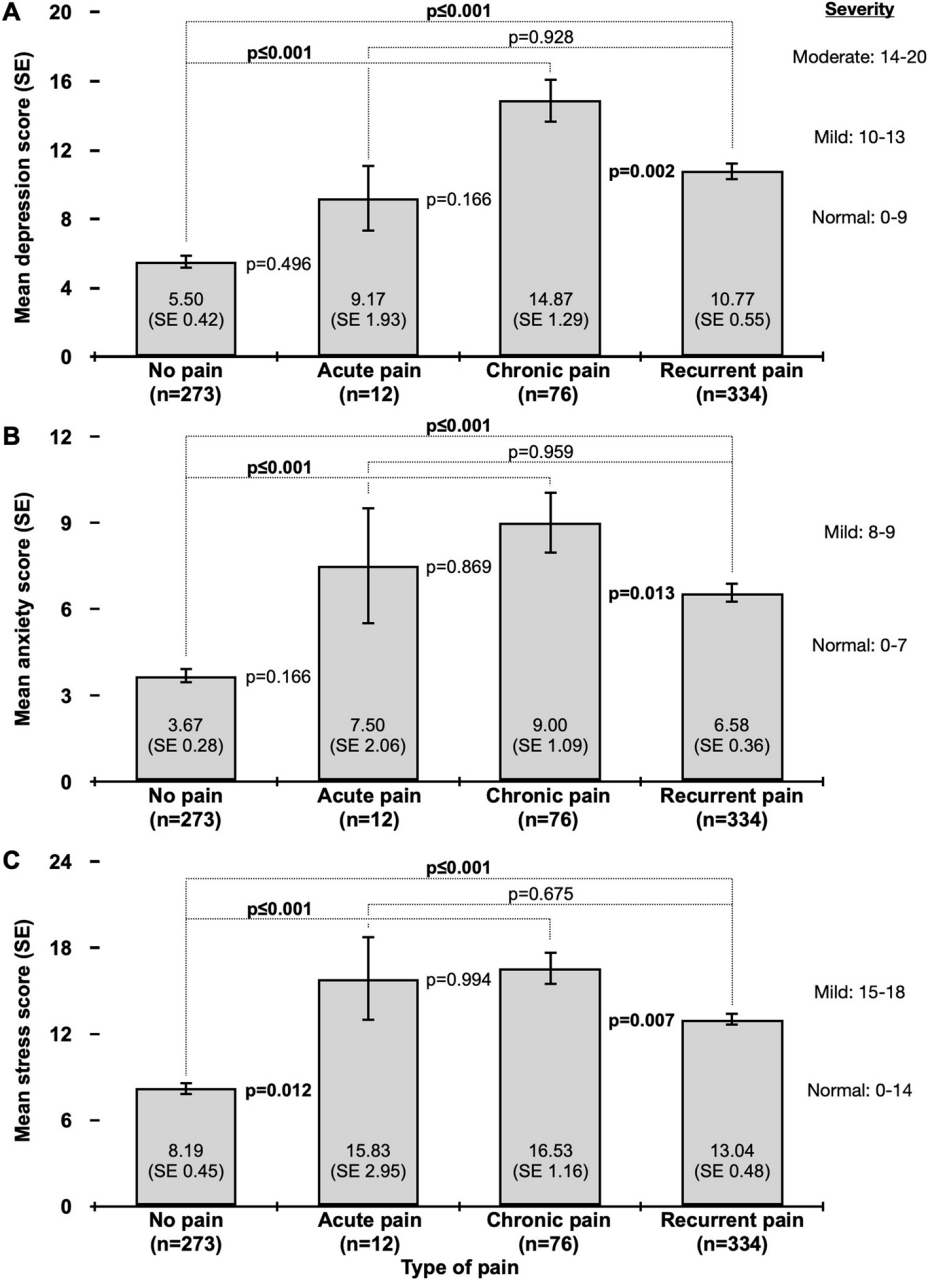
ANOVA post-hoc analysis of doubled DASS-21 sub scale scores for **a** depression, **b** anxiety and **c** stress by type of pain. The *p* values were Tukey corrected and the severity is shown for each doubled DASS-21 sub scale

Scores of the doubled DASS-21 sub scales for the study population (*n* = 695) were 9.11 (SE 0.36) for depression, 5.72 (SE 0.25) for anxiety and 11.56 (SE 0.34) for stress. ANOVAs indicated significant differences in all three sub scale scores (each *p* ≤ 0.001) when compared by type of pain. Post-hoc analysis (Fig. 3) revealed that EMS personnel with chronic pain indicated significantly higher depression scores compared to those with recurrent pain (*p* = 0.002) and no pain (*p* ≤ 0.001), respectively. In addition, study participants with recurrent pain indicated significantly higher depression scores compared to their colleagues without pain (≤ 0.001). Similar results were seen for doubled DASS-21 anxiety score (chronic vs. no pain: *p* ≤ 0.001; chronic vs. recurrent pain: *p* = 0.013; recurrent vs. no pain: *p* ≤ 0.001) and stress score (chronic vs. no pain: *p* ≤ 0.001; chronic vs. recurrent pain: *p* = 0.007; recurrent vs. no pain: *p* ≤ 0.001; acute vs. no pain: *p* = 0.012), respectively. Full detail of the doubled DASS-21 post-hoc analysis is shown in Fig. 3.

## Discussion

This study indicates a high prevalence of pain among participating German pre-hospital EMS personnel with almost 11% suffering from chronic pain and 59% from any type of pain. Of significant importance is that the majority is using freely available analgesics for self-medication, and that those with chronic and recurrent pain indicate higher depression, anxiety, and stress levels.

Previous studies from China, Japan, and Iran reported a prevalence of lower back issues of 12% to 66% in pre-hospital emergency workers (Imani et al. 2018; Okada et al. 2005; Zhang et al. 2019). This is in the range of the present study, identifying pain at lumbar spine in almost 66% of participating pre-hospital EMS personnel with pain (59%), which corresponds to almost 40% of the full study population. Other studies not reporting on EMS personnel only, reported prevalence of lower back pain between 25 and 55% in healthcare workers (Mehrdad et al. 2016; Shaw 2018). Nevertheless, most studies focused on musculoskeletal pain, whereas, our study also included head and gastrointestinal pain. A main cause of pain in pre-hospital EMS personnel is related to the occupation, which is characterized by handling of patients and lengthy periods of standing (Adib-Hajbaghery and Zohrehea 2013; Dunn and Croft 2004; Okada et al. 2005).

To be mentioned here is, that head pain is a frequent event (42%) in German pre-hospital EMS personnel who stated to have any type of pain, with a prevalence comparable to the general German population (female 57.5% / male 44.4%). Nevertheless, migraine (female 14.8% / male 6.0%) and tension headache (female 10.3% / male 6.5%) (Porst et al. 2020), which are by definition most comparable to recurrent pain in our study, indicate lower prevalence in the general population compared to head pain in pre-hospital EMS personnel with recurrent pain (43.42%; Table 2).

More than half of pre-hospital EMS personnel disclose to use analgesics. The majority indicated an intake of freely available Ibuprofen, with a trend of analgesics with higher potency being more frequently used by participants with chronic pain. The most frequently used analgesics in the general German population with migraine are Ibuprofen, Paracetamol, Acetylsalicylic acid, Metamizole, Triptanes, Diclofenac, Naproxen and Tramadol (Porst et al. 2020), which is to some extent comparable to the analgesics used by pre-hospital EMS personnel with pain in the present study (Fig. 2). Few other studies are published on the usage of substances in healthcare workers, which are often regarding misuse (Baldisseri 2007; Bennett and O’Donovan 2001; Merlo et al. 2013). But to the best of our knowledge, no recent data are available specifically focusing on analgesic intake by pre-hospital EMS personnel, but the high number of analgesic users in this population with pain is an alarming issue. Especially the circumstance that study participants with chronic pain are also using analgesics with a high addiction potential like Tramadol or Tilidine. These two opioids are often subject to misuse and Krüger et al. reported that Tramadol was the most and Tilidine the fourth most frequently mentioned opioid in a German-speaking internet forum for drugs, which the authors analyzed regarding misuse (Krüger et al. 2014).

Besides musculoskeletal diseases, previous studies have reported that pre-hospital emergency workers are often suffering from mental disorders (Berger et al. 2012; Sterud et al. 2006; Van Der Ploeg and Kleber 2003). The overall depression, anxiety and stress levels in the present study were in the normal severity range. But our study also shows that pre-hospital EMS personnel with chronic and recurrent pain, indicated significantly higher depression, anxiety and stress levels compared to participants with no pain (Fig. 3). Previous studies indicated that the workplace, work-related stressors, social conflicts and exposure to critical incidents are important in terms of mental health issues (Carleton et al. 2020; de Boer 2011; Donnelly et al. 2016; Hruska and Barduhn 2021; Leszczyński et al. 2019). Donnelly et al. (2016) showed that posttraumatic stress symptomatology is significantly associated with operational (e. g. shift work, fatigue, risk of getting injured) and organizational stress (e. g. conflict with supervisors, changes in policies) in paramedics. But there are also known associations between pain and depression, suggesting that relieving pain may relieve depression and vice versa (Angst et al. 2020). It is also reported, that pain and depression correlate in terms of brain regions and neurological function system (Sheng et al. 2017), which might explain a potential link between mental health and pain.

Important to mention here is, that study participants with chronic pain showed a moderate level of depression and suffered from higher pain intensities. In addition, they are more vulnerable to the usage of Tramadol or Tilidine, and mental health disorders. Therefore, pre-hospital EMS personnel with chronic pain, but also recurrent pain, need to be identified and supported to preserve long-term health and to avoid inability to work or drug dependency.

This study has several limitations which will be discussed in the following.

First, our study sample has to be considered small (*n* = 774), since 71,000 people are working in the pre-hospital emergency service in Germany (HPA 2021). In addition, we cannot exclude, that EMS personnel with pain were more accessible for the topic of this study and consequently participated more frequently compared to those without pain. Therefore, our study is not necessarily representative for all pre-hospital EMS workers in Germany and more studies are needed to determine a more exact prevalence of pain, analgesic intake and mental health status.

Second, some federal states were overrepresented by study participants such as Bavaria (19.51%) or Rhineland-Palatinate (18.86%) compared to highly underrepresented federal states such as Thuringia or Mecklenburg-Western Pomerania (each 0.39%) (Table 1). According to this and the low number of participants the representativeness of our study has to be considered limited.

Third, data was collected using an online survey tool and we had to rely on the honesty of the study participants.

Fourth, this study was conducted during the corona pandemic. Therefore, we do not know if the pandemic itself, measures against the virus or a higher pandemic-related workload had an influence on depression, stress and anxiety level or prevalence of pain in the study participants. Based on the data of the Robert Koch Institute, the number of daily reported COVID-19 cases increased from 4679 to 22,288 during the study period. If we also consider the three months before start of our study, the daily number of cases ranged from 131 to < 1000 per day in July, from 301 to < 1800 in August and 579 to < 2800 in September (RKI 2021), respectively. Due to the low number of cases during the 3 months before start of our study, we do not expect that the pandemic strongly affected the prevalence of chronic pain in our study sample. Nevertheless, the strong increase in daily COVID-19 cases during the study period might have influenced the results on recurrent pain and in particular strongly those on acute pain. For the evaluation of the effect of pain on mental health, we can consider the study population with no pain as a control group, since they should have been mentally affected by the pandemic to a similar amount like the three pain populations.

Fifth, even though our results indicate significantly higher DASS-21 subscale scores in study participants with chronic / recurrent pain, one has to take into account that other factors can influence mental health. Therefore, future research similar to the present study should include questionnaire items on previous diagnosed mental health disorders, physical disorders and more sociodemographic and work-related factors (e. g. income, family status, education, workplace, work-related stressors) with known impact on mental health. Furthermore, other study designs such as the retrospective analysis of health insurance data might allow even more objective insights into the health status of persons working in the pre-hospital EMS. This kind of data, which usually includes sociodemographic factors and previous diagnosis, represents an appropriate data base to identify the relationship between pain and mental health in this particular group of workers.

## Conclusion

This study indicates a high prevalence of pain in participating German pre-hospital EMS personnel, with more than 50% suffering from chronic or recurrent pain. Consequently, usage of analgesics is high and every second emergency personnel with pain is taking analgesics. In addition, pre-hospital EMS personnel with chronic or recurrent pain indicated significantly higher scores for mental disorders. Nevertheless, it is unclear if their pain is the main cause for the increased depression, anxiety or stress levels. Notwithstanding the above, results indicate several health issues in German pre-hospital EMS personnel. Measures to prevent musculoskeletal and mental disorders are required, which are matching the day-to-day work and workload of this specific group of healthcare workers.
